# Hydrophilic Graphene Preparation from Gallic Acid Modified Graphene Oxide in Magnesium Self-Propagating High Temperature Synthesis Process

**DOI:** 10.1038/srep35184

**Published:** 2016-10-11

**Authors:** Lei Cao, Zhenhuan Li, Kunmei Su, Bowen Cheng

**Affiliations:** 1 State Key Laboratory of Separation Membranes and Membrane Processes, School of Materials Science and Engineering, Tianjin Polytechnic University, 300160 Tianjin, China

## Abstract

Hydrophilic graphene sheets were synthesized from a mixture of magnesium and gallic acid (GA) modified graphene oxide (GO) in a self-propagating high-temperature synthesis (SHS) process, and hydrophilic graphene sheets displayed the higher C/O ratio (16.36), outstanding conductivity (~88900 S/m) and excellent water-solubility. GO sheets were connected together by GA, and GA was captured to darn GO structure defects through the formation of hydrogen bonds and ester bonds. In SHS process, the most oxygen ions of GO reacted with magnesium to prevent the escape of carbon dioxide and carbon monoxide to from the structure defects associated with vacancies, and GA could take place the high-temperature carbonization, during which a large-area graphene sheets formed with a part of the structure defects being repaired. When only GO was reduced by magnesium in SHS process, and the reduced GO (rGO) exhibited the smaller sheets, the lower C/O ratio (15.26), the weaker conductivity (4200 S/m) and the poor water-solubility because rGO inevitably left behind carbon vacancies and topological defects. Therefore, the larger sheet, less edge defects and free structure defects associated with vacancies play a key role for graphene sheets good dispersion in water.

Graphene is a novel 2-D carbon nano-material, and it is made up of conjugated sp^2^ carbons and arranged in a honeycomb structure, which makes graphene to display the excellent electrical, mechanical, thermal and optical properties^1^,^2^,^3^,^4^,^5^,^6^,^7^. Graphene can be prepared by physical or chemical methods, such as chemical vapor deposition (CVD), ultrasonic exfoliation of graphite, epitaxial growth and graphene oxide (GO) reduction^8^,^9^,^10^,^11^,^12^,^13^,^14^,^15^. The epitaxial growth and CVD methods can be used to provide the large area and almost free-defect graphene sheets for applications in electronics^7^, but those free-defect graphene sheets were difficult to be mass-produced.

Known as GO rich in oxygen containing groups such as hydroxyl, carbonyl and epoxy group, and the chemical reduction of GO is the most efficient method for low-cost and large-scale production of graphene^12^,^13^,^14^,^15^. Hydrazine^13^, NaBH_4_^14^ and ascorbic acid^15^ *et al*. had been used to reduce GO, and hydrazine and NaBH_4_ were effective in removing oxygen of GO. When N_2_H_4_ was used as reducing agent, N can be doped in the as-prepared graphene. Although NaBH_4_ showed excellent ability in GO reduction, the hydrolysis property made it difficult to get a stable NaBH_4_ aqueous solution^16^. Furthermore, it is usually considered that it is difficult to form large area graphene or free-defect sheets though chemical reduction^17^. The high electrical conductivity (56500 S/m) of graphene was obtained via thermal exfoliation of GO at low temperature and then annealing at 1900 °C under vacuum conditions^18^. Graphene also can be prepared by magnesium thermal reduction of GO with annealing at 650 °C for 6 h in Ar atmosphere^19^, but the high temperature reduction of GO under inert atmosphere condition leads to the number of CO or CO_2_ elimination from the carbon lattice to form the atomic vacancies or graphene fragment, which cause the structural deterioration of as-prepared graphene^20^. Most importantly, graphene prepared from GO reduction and annealed at high temperature all exhibited the high hydrophobic property.

SHS represents an attractive alternative to conventional methods for materials synthesis^21^,^22^,^23^. This technique, which involves self-propagating reactions of either solid-solid or gas-solid type, is characterized by the fact that, once the starting mixture is ignited by means of external thermal sources for relatively short times, highly exothermic reactions propagate through the mixture in the form of self-sustained combustion wave, progressively leading to final products without requiring additional energy. Reaction characteristics include the high rates of the combustion front (10^–4^~10^−3 ^m/s), generation of high temperatures (1700~2000 °C, even to 4000 °C), and rapid heating (10^3^~10^4^ K/s). The SHS technique is also characterized by process simplicity, short reaction time, easy-to-build equipment, low energy requirements, and the possibility of obtaining complex or metastable phases. GA has three hydroxyl and one carboxyl groups in its benzene ring which has the same oxygen-containing groups with GO. The network holes of GO can be darn with GA by hydrogen bonds and ester bonds, and GO sheets can also be connected together by gallic acid. In this paper, GO was modified by GA to form GO_GA_, and GO_GA_ was further reduced by magnesium in SHS process under air atmosphere condition (Fig. 1). This environmentally friendly technology is easy to achieve the higher C/O ratio, high conductivity and excellent water-solubility graphene sheets.

## Results

In order to discuss the effects of addition amount of GA on sheet area, smooth, edge defects and hydrophobic property of graphene sheets, 1.0 g, 1.5 g and 2.0 g GA were loaded into equal mass of GO solution, and then reduced by magnesium with the same methods. The resulting materials are denoted as rGO_GA1_, rGO_GA_ and rGO_GA2_, respectively.

**Figure 2:**
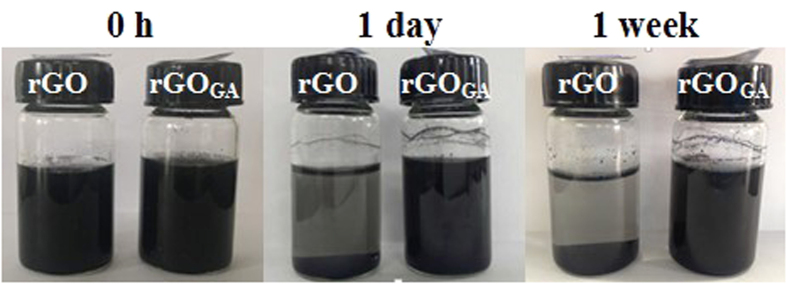
Dispersions of rGO and rGO_GA_ in aqueous solution at different time intervals (~1mg/mL).

**Figure 3:**
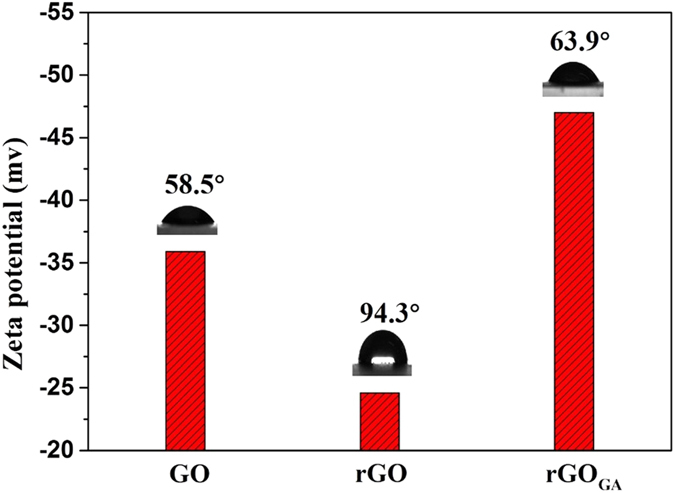
Zeta potential and contact angle of GO, rGO and rGO_GA._

The water solubility of rGO and rGO_GA_ were investigated in deionized water at different time intervals. It can be seen from Fig. 2, rGO has lower dispersibility in aqueous solution, but rGO_GA_ can be uniformly dispersed in aqueous solution for a long time. Zeta potential is a direct parameter on reflecting the interaction among suspended particles. The zeta potentials of aqueous GO, rGO and rGO_GA_ were −35.9 mV, −24.6 mV and −47.0 mV, respectively (Fig. 3). Particles with zeta potentials more negative than −30 mV are considered to form stable suspension due to interparticle electrostatic repulsion^24^,^25^. Therefore, rGO precipitated within one day, and rGO_GA_ dispersed in aqueous solution to form a stable colloidal for long time, indicating the excellent water-solubility of rGO_GA_. But beyond that, contact angle measurements were used to further prove the hydrophilic of rGO_GA_. It was noticed that the contact angle of the GO, rGO, and rGO_GA_ were 58.5°, 94.3° and 63.9°, respectively. And compared rGO_GA_, the contact angle of rGO_GA1_ and rGO_GA2_ were 76.4° and 96.3° (Figure S1). The test results proved once again that the excellent hydrophilicity of rGO_GA_.

**Figure 4:**
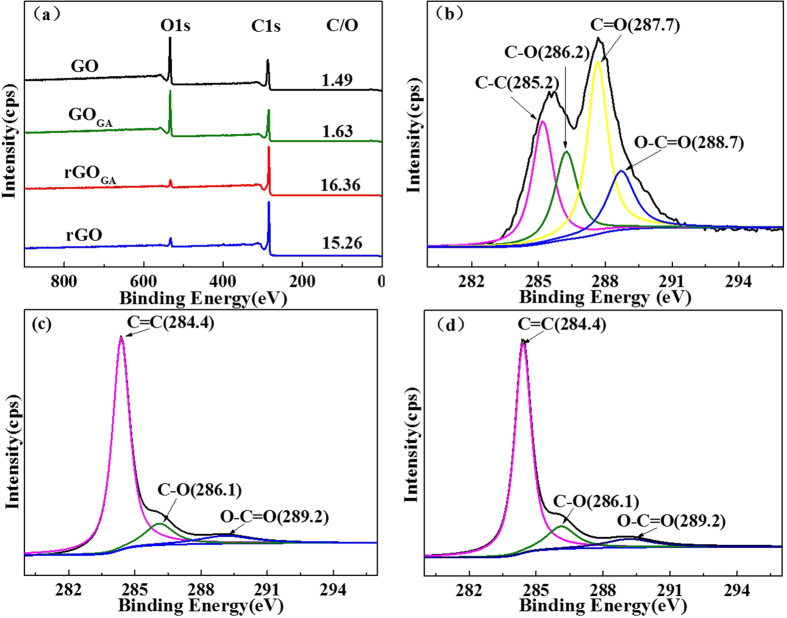
XPS survey spectra (**a**), C1s XPS spectra of GO (**b**), rGO_GA_ (**c**) and rGO (**d**).

In order to investigate the reasons for excellent water-solubility of rGO_GA_, XPS was used to investigate the chemical structure and composition of the prepared samples. Only C1s and O1s peaks can be observed in the XPS survey scan (Fig. 4a), and no hetero atoms (including Mg) were doped into graphene. As for rGO_GA_ and rGO, the intensity of the O1s band dramatically decreases and the intensity of the C1s band significantly increases, suggested that oxygen-containing groups had been effectively removed. The C/O atomic ratio of GO and GO_GA_ are 1.49 and 1.63. However, the C/O atomic ratio of rGO_GA_ and rGO increased to 16.36 and 15.26, indicating the full reduction of GO_GA_. Most importantly, C/O atomic ratio of rGO_GA_ and rGO is much higher than that of GO reduced by other chemicals, such as hydrazine (10.3)^13^, NaBH_4_ (8.6)^14^ and LiAlH_4_ (12)^26^. C1s XPS spectra of GO (Fig. 4b) showed a peak at 285.2 eV that corresponded to C-C bonds of carbon atoms in a conjugated honey-comb lattice^27^, and other three different peaks centered at 286.2 eV, 287.7 eV and 288.7 eV are also observed, corresponding to single or double bonds in aromatic rings, i.e. C-O (epoxy and alkoxy), C=O, and O-C=O groups, respectively. Figure 4c,d showed that C=O groups completely disappeared in rGO_GA_ and rGO, indicating that the delocalized π-conjugated structure was well restored in rGO_GA_ and rGO^28^. Importantly, the C/O atomic ratio of rGO_GA_ was higher than that of rGO, but rGO_GA_ displayed much better hydrophilic than rGO. Those results are far beyond our expectations. A hypothesis was proposed that graphene composition is not the main factor to influence the hydrophilic or hydrophobic property, but its structure characteristics must play a key role on its hydrophilic or hydrophobic property.

**Figure 5:**
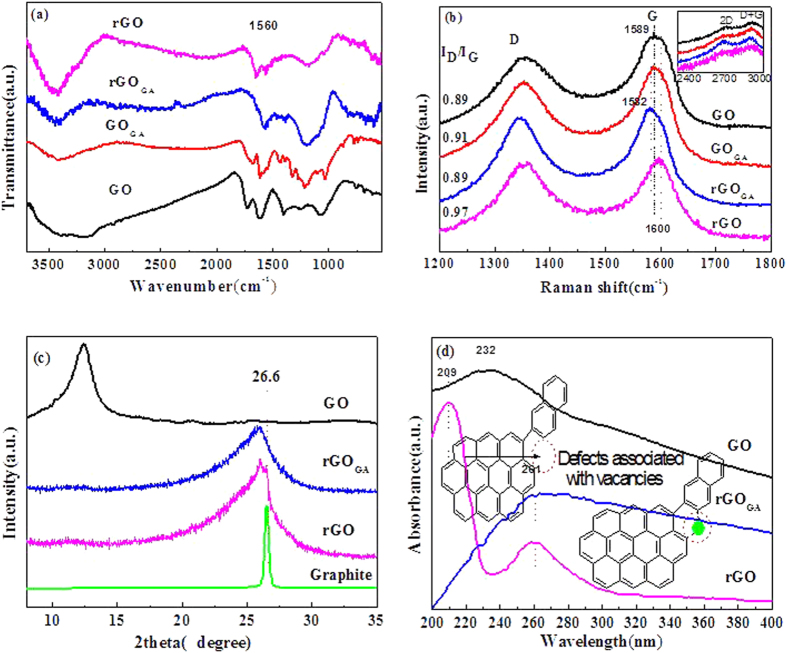
(**a**) ATR-IR spectra of GO, GO_GA_, rGO_GA_, and rGO. (**b**) Raman spectra of GO, GO_GA_, rGO_GA_, and rGO. (**c**) XRD patterns of graphite, GO, rGO_GA_ and rGO powder. The 2θ angles of the XRD peaks (d-spacing) of GO and rGO_GA_ shifted from 12.4° (d-spacing ~0.8 nm) to 25.8° (d-spacing ~0.34 nm) after reduction. (**d**) UV-vis absorption spectra of GO, rGO_GA_ and rGO.

Figure 5a showed the FT-IR spectra of GO, GO_GA_, rGO_GA_ and rGO. A series of absorption bands ranging from 1000 to 1750 cm^−1^ exhibited the presence of C=O bonds (at 1720 cm^−1^), O–H bending (at 1621 cm^−1^), O-H deformation (at 1406 cm^−1^), C-O groups in epoxy (at 1221 cm^−1^) and C-O groups in alkoxy (at 1057 cm^−1^)^29^,^30^. Hydroxyl (OH) groups have an intensive absorption band between 3200 and 3700 cm^−1^, due to the presence of moisture intercalated within hydrophilic GO sheets^30^. After modified with gallic acid (GO_GA_), the characteristic bands for oxygen containing groups did not change remarkably, but some new FT-IR absorptions of GO_GA_ appeared in the range of 1490–1190 cm^−1^, which indicated that gallic acid molecules were adsorbed or darned on GO^25^. It is noteworthy that the C=O band of GO_GA_ is shifted by 44 cm^−1^ from 1720 cm^−1^ to 1676 cm^−1^ compared to GO, corresponding to the formation of O=C-O-C groups, which demonstrates that a portion of gallic acid are combined with GO by esterification. After samples undergo magnesium self-propagating high temperature synthesis process, the characteristic bands for oxygen-containing groups of rGO and rGO_GA_ almost completely disappeared, but the bands of aromatic carbon (smaller π-conjugated C=C structure) at 1575 cm^−1^ and (Larger π-conjugated C=C structure) at 1560 cm^−1^ appeared. These observations suggested that GO and GO_GA_ were well reduced, and the larger-area π-conjugated structure was established in rGO_GA_.

GO, GO_GA,_ rGO_GA_ and rGO were further characterized by Raman spectroscopy. As shown in Fig. 5b, there are two significant peaks for these four samples, assigned to D band (~1350 cm^−1^) and G band (~1585 cm^−1^), which are E2g vibration mode in-plane and A1g breathing mode^31^. The appearance of a distinct D band in the spectrum is an indication of disorder in graphene originating from the defects associated with vacancies and grain boundaries^32^. As compared to the G band of GO, that of rGO_GA_ was shifted by 7 cm^−1^ from 1589 to 1582 cm^−1^, displaying a high reduction degree^33^. Previous reports exhibited that the size of the defect-free sp^2^ cluster regions is the inverse of the ratio of the D and the G band integrated intensities (I_D_/I_G_)^32^,^34^,^35^. In the case of GO, the I_D_/I_G_ ratio was 0.89, and the I_D_/I_G_ ratio of GO_GA_ increased to 0.91. However, I_D_/I_G_ ratio of rGO was 0.97, rGO_GA_ I_D_/I_G_ ratio declined to 0.89. From those results, the following conclusions can be drawn: (1) the most oxygen ions of GO reacted with magnesium to form MgO, reducing the number of carbon atoms vacancies of the carbon lattice; (2) the average size of the sp^2^ domains of rGO_GA_ was larger than rGO slightly; (3) gallic acid can repair the structure defects of graphene during magnesium thermal reaction. Apart from D band and G band, there are two Raman bands with weaker intensity called 2D and D + G located at 2680–3000 cm^−1^ as shown in insets of Fig. 5b. Zhan *et al*.^28^ discovered that it could be utilized to distinguish the electronic conjugation of GO and rGO_GA_ by comparing these two bands. Compared with rGO (or GO), the enhancement of I_2D_/I_D+G_ ratio of rGO_GA_ can be clearly observed, which suggests that the full recovery and the larger-area π-conjugated structure for rGO_GA_.

XRD was used to characterize graphite, GO, rGO_GA_ and rGO. Figure 5c shows the 2θ peak of graphite powder was at 26.6° (d-spacing ~0.34 nm) and a broad peak near 12.4° (d-spacing ~0.80 nm) was observed for GO, indicating that the graphite was fully oxidized. Compared with the parent GO, the peak of rGO_GA_ shows an obvious shift to higher 2θ angles (25.8°; d-spacing ~0.35 nm), suggesting that rGO_GA_ was well ordered with two-dimensional sheets and well removal of surface functional groups^11^. Compared to rGO_GA_, a weak shoulder peak at 26.5° of rGO was found, which revealed a small portion of the reduced graphene sheets agglomerated together or formed graphite structure^36^.

Samples were also characterized by UV-vis absorption, as shown in Fig. 5d, GO has a intensive absorption band at 235 nm, which was due to the π→π* transition of aromatic C=C bond. In addition, a weaker absorption shoulder at around 303 nm, which was attributed to the n→π* transition of the C=O bond^37^,^38^,^39^,^40^. The π→π* transition band of rGO_GA_ and rGO red shifted to 264 nm owing to the restoration of the conjugated structure, which indicated the removal of oxygen-containing functional groups and the disappearance of n→π* transition band^41^,^42^. However, some π→π* transition band of rGO shifted to 209 nm, indicating the defects associated with vacancies. Those results further proved that GA took place the high-temperature carbonization to repair the structural defects during SHS process, therefore, a large-area graphene sheets of rGO_GA_ formed with a part of the structure defects being repaired during a SHS process.

**Figure 6:**
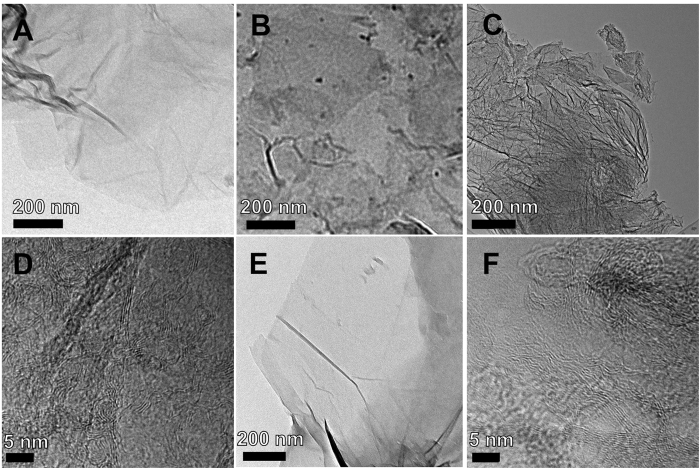
TEM images of GO (**A**), GO_GA_(**B**), rGO (**C**) and rGO_GA_ (**E**). High resolution TEM images of rGO (**D**) and rGO_GA_ (**F**).

Figure 6A,B showed the TEM images of GO and GO_GA_. By comparison, GA was adsorbed on the surface and edge of GO by the interaction between GA molecule and defects of GO (Fig. 6B). Figure 6C,E showed the TEM images of few-layer rGO and rGO_GA_. There are some wrinkles and scrolls in rGO sheets like the reported work^25^, and the TEM analysis of rGO showed the edge of rGO sheets generated irregular defect with the formation of small fragment. However, rGO_GA_ showed a larger sheet, nearly no wrinkles and scrolls, and less edge defects. In addition, Figure S2A,b showed the TEM images of few-layer rGO_GA1_ and rGO_GA2_. Due to little addition amount of GA, it still can be seen that small number of the edge and inside defects from Figure S2A. It can be shown that only parts of graphene defects were restored. In contrast to rGO_GA1_, Figure S2A showed that there were more carbon impurities which were obtained by magnesium thermal reduction of excess GA on the surface of graphene sheets. The high-resolution TEM (Fig. 6D,F) clearly exhibits the signature image of the few-layer graphene with the number of layers ranging from 3 to 8. The measured lattice space of this material is about 3.45 Å, which is in good agreement with the thickness of a monolayer graphene. The biggest difference between Fig. 6D,F are that the small layers of rGO are messy accumulation, but the large layers of rGO_GA_ are in an ordered arrangement because of the restoration of GA. These differences are attributed to the generation of atomic vacancies in the rGO sheets, and the atomic vacancies had a high mobility at high temperature. The amount of generated atomic vacancies was so large that rGO sheets disintegrated and caused a significant structural deterioration at high temperature^20^. Several experimental and modeling studies have focused on exploiting surface roughness to engineer super hydrophobicity^43^,^44^. It is generally accepted that roughness-induced hydrophobicity. rGO_GA_ has a highly polished surface because of the larger sheet, smooth, no fold, less edge defects. So it shows the more hydrophobic property. Based on those characterizations, a conclusion can be drawn that the wrinkles, scrolls, irregular structure defects were associated with vacancies and irregular grain boundaries rather than the C/O atomic ratio to determine the hydrophilic or hydrophobic property of graphene sheets. In summary, the larger sheet, smooth, no fold, less edge defects and the structure defects associated with vacancies play a key role for graphene sheets good dispersion in water media.

**Figure 7:**
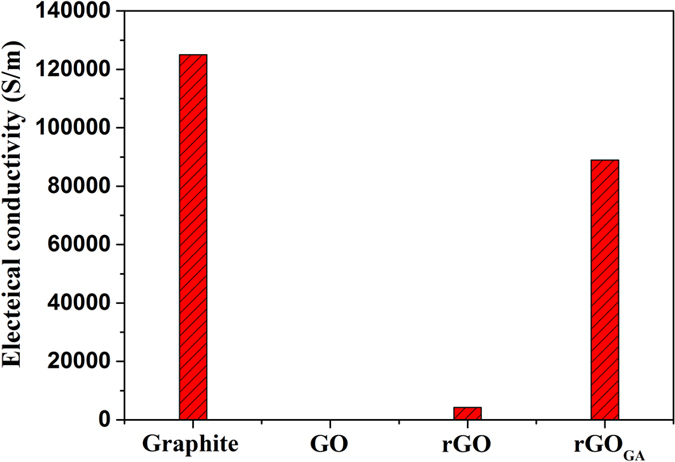
The electrical conductivity of different samples.

The electrical conductivity is considered a highly sensitive indicator of the extent to which electronic conjugation is restored after deoxygenation of GO. The electrical conductivity of compressed-powder samples of the pristine graphite, GO, rGO and rGO_GA_ were measured at room temperature (Fig. 7), and the electrical conductivity are 125000 S/m, 0.012 S/m, 4200 S/m and 88900 S/m, respectively. The conductivity of rGO_GA_ is much high than that of rGO, and closely approaches that of pristine graphite. In addition, the electrical conductivity of rGO_GA1_ and rGO_GA2_ are 29760 S/m and 59277 S/m, respectively (Figure S3). Conclusion can be drawn that the numerous defects associated with vacancies and carbon impurities in graphene sheets play the key role on the lower electrical conductivity of grapheme.

**Figure 8:**
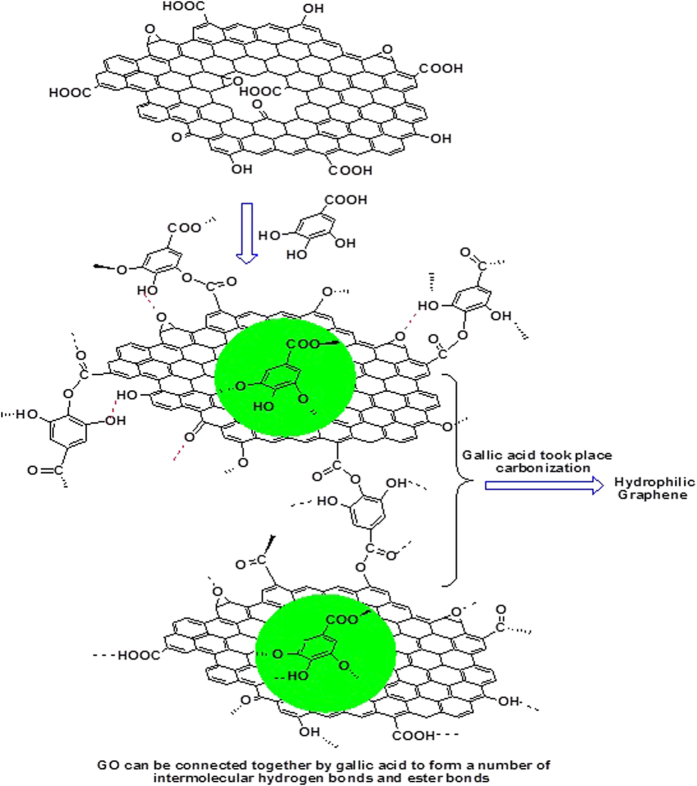
Suggested mechanisms for the modification of gallic acid and the reduction of GO_GA_ with Gallic acid-assisted magnesium reduction of GO in a SHS process.

The following mechanism was suggested for our reduction process (Fig. 8). Gallic acid and GO can be connected together by forming a number of intermolecular hydrogen bonds. Importantly, Gallic acid and GO can take place the esterification reaction at 90 °C for 24 h. Therefore, gallic acid can act as the medium to connect together with adjacent GO sheets though ester bond and hydrogen bonds. After gallic acid took place the high-temperature carbonization during magnesium high temperature thermal reduction process, a part of pyrolytic carbon self-assemble into a graphene structure, and another portion of carbon filled inside of the graphene atomic vacancies to repair of structural defects. Magnesium thermal reaction is a classical reaction which is usually used to reduce metal oxide. Magnesium can capture oxygen atoms of metal oxide to generate corresponding metal element and magnesium oxide. Importantly, previous article reported that burning magnesium metal in dry ice resulted in few-layer nanosheets of graphene in high yields^45^. In other words, Magnesium also can capture the oxygen atom of carbon dioxide. So during the SHS process, magnesium could capture the oxygen atom from GO, which also prevent the escape of carbon dioxide and carbon monoxide to from the structure defects associated with vacancies. Therefore, a large-area hydrophilic graphene sheets were formed by a SHS process.

## Conclusion

In conclusion, a novel and facile approach to the synthesize graphene sheets based on magnesium SHS process is reported using GO modified by gallic acid (GO_GA_) as precursor. The merit of this method is that the process carried out in the air atmosphere without external heating source, and gallic acid can repair the structure defects of graphene during SHS process. The obtained graphene sheets could be stably dispersed in water for more than one month. The wrinkles, scrolls, irregular structure defect associated with vacancies and grain boundaries (rather than C/O atomic ratio) determine the hydrophilic property. While the less edge defects and free structure defects associated with vacancies play a key role for graphene sheets good dispersion in H_2_O. Moreover, the synthetic process can be utilized to produce large-scale graphene by an environmentally friendly way. Obviously, these advantages will facilitate its industrial applications.

## Materials and Methods

### Materials

Natural graphite powder (80 mesh) was supplied by Tianjin Chemical Co., Ltd and used as received. NaNO_3_ (99.8%), KMnO_4_ (99.5%), H_2_SO_4_ (98%), HCl (35%), magnesium powder and magnesium ribbon *et al*. were purchased from Tianjin Kermel Chemical Reagent Co., Ltd.

### Characterization

FT-IR was recorded from a TESOR 37 (BRUKER Corporation, Germany) and operated by attenuated total reflectance (ATR) in the wavenumber range of 4000–500 cm^−1^. Raman spectra were obtained by using a micro-Raman system on (Renishaw, RW1000-In Via) with an excitation energy of 2.41 eV (l = 514.5 nm). XRD patterns were measured on a Bruker XPS (Germany) using Cu Kα radiation. UV-vis detection was carried out by UV/Vis Spectrometer (Thermofisher, USA). TEM and HRTEM images were obtained from a JEM-2100 transmission electron microscope (JEOL, Japan). Electrical conductivity of samples was measured on a ST2722 semiconductor powder resistivity tester (Suzhou, China). X-ray photoelectron spectroscopy (XPS) analysis was carried by (Thermofisher, K-alpha).

### Sample preparation

#### Preparation of GO

GO was prepared by modified Hummers’ method^45^. 5.0 g graphite, 2.5 g NaNO_3_, and 125 ml H_2_SO_4_ were loaded in a 1000 ml round-bottom flask with magnetic stirring. After being cooled with ice-water bath at 0 °C for 1 h, 17.5 g KMnO_4_ was added into above reaction system. After reaction was kept at 0–5 °C for 1 h and then heated at 35 °C for 2 h, the mixture was diluted with 250 ml deionized water and reacted at 98 °C for 45 min. 200 ml deionized water and 50 ml H_2_O_2_ (30%) were added into above suspension under the condition of magnetic stirring. After centrifugation and dry, GO was obtained.

#### Preparation of GO_GA_

Exfoliation of GO was obtained by ultrasonication in water for 2 h. GO_GA_ was prepared in three steps: (1) 1.5 g gallic acid was loaded into 500 ml GO (5 mg/ml) solution, and the mixture was dispersed by ultrasound for 40 min. (2) the mixed solution was allowed to stand at room temperature for 3 h. (3) The mixed solution was dried at 90 °C under air atmosphere for 24 h to obtain the mixture of gallic acid-modified GO (GO_GA_). As a control, different addition amount of gallic acid (1.0 g and 2.0 g) in equal mass of GO (were labeled as GO_GA1_ and GO_GA2_) were prepared with the same method.

#### Preparation of rGO_GA_, rGO_GA1_, rGO_GA2_ and rGO

3.0 g Magnesium powder and 1.5 g GO_GA_ powder were mixed evenly. The mixture was put in a crucible, and mixture was covered with a thin layer of magnesium powder on the surface of mixture. Mixture was ignited by magnesium ribbon under air atmosphere condition. After the reaction is over, the mixed products were transferred to a beaker containing 250 ml of 3 M HCl. Products were transferred into the acid solution to remove MgO and residual Mg. The resulting rGO_GA_ was collected with filtration, washed with deionized water until the filtrate turned out to be of neutral. Obtained rGO_GA_ was dried at 60 °C under high vacuum conditions for 10 h. As a control, rGO_GA1_, rGO_GA2_ and rGO were prepared from GO_GA1_, GO_GA2_ and GO reduction by magnesium with the same methods.

#### Contact angle measurement

The contact angle of the samples was measured on DSA 100 (KRUSS) contact angle system at ambient temperature. Before the measurement, 20 mg power samples were dispersed in 500 ul ethanol with ultrasonic treatment 1 h. In the following, the suspension was dropped onto a glass slide to form a uniform film.

#### Electrical conductivity measurement

Electrical conductivity of samples was measured on a ST2722 semiconductor powder resistivity tester (Suzhou, China). The powder sample was filled into a test slot, then applying a pressure about 18 MPa. The electrical resistivity tester would reflect the electrical resistivity of the sample directly. The electrical conductivity of samples was calculated through the following equations:


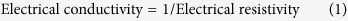


## Additional Information

**How to cite this article**: Cao, L. *et al*. Hydrophilic Graphene Preparation from Gallic Acid Modified Graphene Oxide in Magnesium Self-Propagating High Temperature Synthesis Process. *Sci. Rep.* **6**, 35184; doi: 10.1038/srep35184 (2016).

## Supplementary Material

Supplementary Information

## Figures and Tables

**Figure 1:**
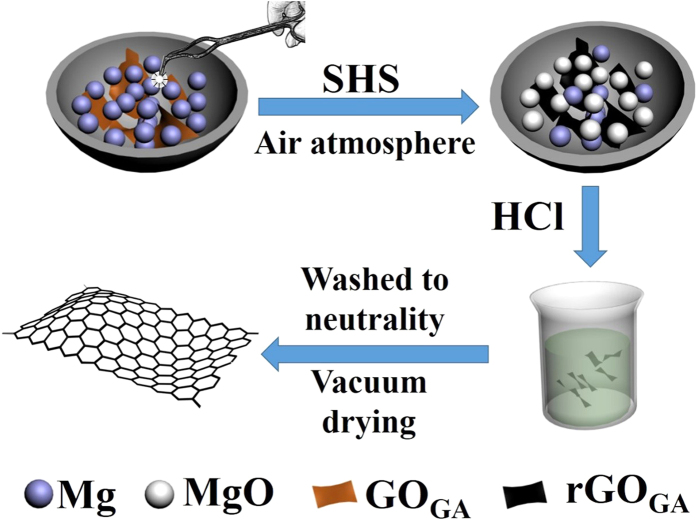
GO_GA_ reduction by Mg in SHS process.
