# Supplementary material for: Hydrophilic Graphene Preparation from Gallic Acid Modified Graphene Oxide in Magnesium Self-Propagating High Temperature Synthesis Process
Source: Sci Rep. 2016 Oct 11;6:35184. doi: 10.1038/srep35184 (PMC5057088; doi:10.1038/srep35184)
Supplement: Supplementary Information [file srep35184-s1.pdf]

Supplementary Information

Hydrophilic Graphene Preparation from Gallic Acid Modified Graphene Oxide in Magnesium Self-Propagating High Temperature Synthesis Process

Lei Cao, Zhenhuan Li*, Kunmei Su, Bowen Cheng

State Key Laboratory of Separation Membranes and Membrane Processes, School of Materials Science and Engineering, Tianjin Polytechnic University, 300160 Tianjin, China.

***Corresponding Authors**

Tel: +86 022 83955358; fax +86 022 83955055;

e-mail: zhenhuanli1975@aliyun.com or lizhenhuan@tjpu.edu.cn

In order to discuss the effects of addition amount of GA on sheet area, smooth, edge defects and hydrophobic property of graphene sheets, 1.0g, 1.5g and 2.0g GA were loaded into equal mass of GO solution, and then reduced by magnesium with the same methods. The resulting materials are denoted as rGO_{GA1}, rGO_{GA} and rGO_{GA2}, respectively.

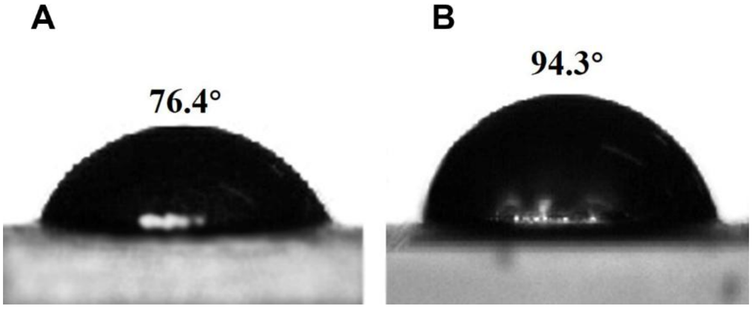

Figure S1. contact angle of rGO_{GA1} and rGO_{GA2}.

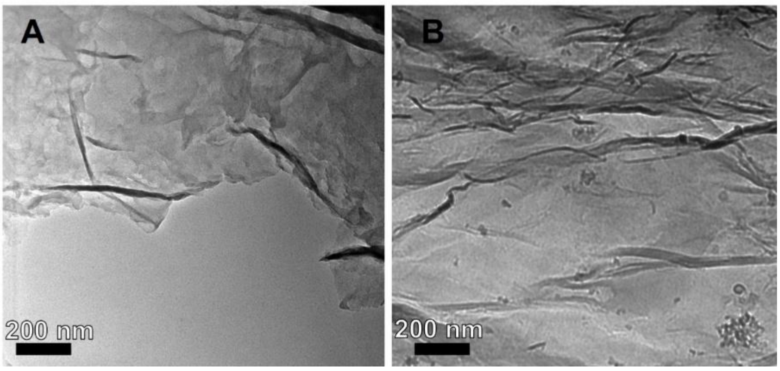

Figure S2. TEM images of rGO_{GA1} and rGO_{GA2}.

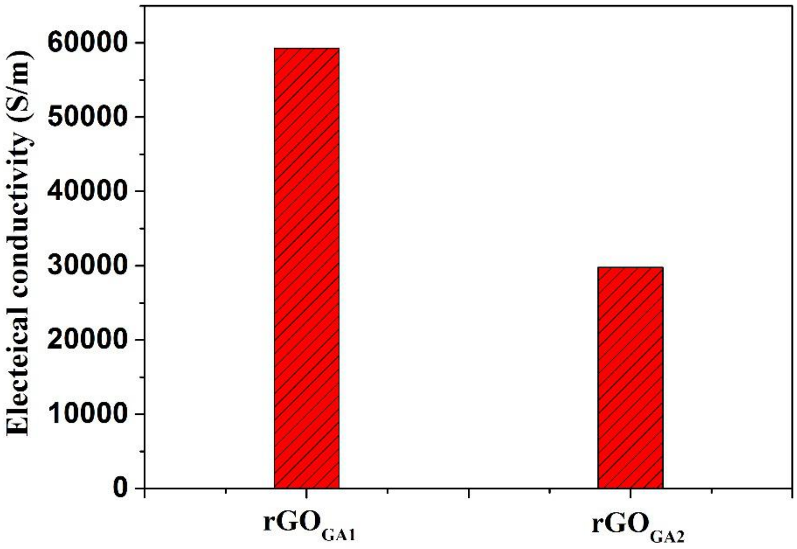

Figure S3. The electrical conductivity of rGO_{GA1} and rGO_{GA2}.
